# Managing quality-oriented big data: a conceptual architecture for acquisition, fusion, and analytics in data-driven organizations

**DOI:** 10.3389/fdata.2026.1977331

**Published:** 2026-10-16

**Authors:** Shahlokhon Gafurova, Shukhrat Rahmatov, Umida Zakhidova, Dilnoza Rakhimova, Gulshan Kabilova

**Affiliations:** 1 Department of Economics, Bukhara State University, Bukhara, Uzbekistan; 2 Department of Scientific Research, Innovation and Training of Scientific and Pedagogical Personnel, Bukhara State Technical University, Bukhara, Uzbekistan; 3 Department of Economics and Management, Bukhara State Technical University, Bukhara, Uzbekistan; 4 Bukhara State Medical Institute, Bukhara, Uzbekistan

**Keywords:** big data management, data fusion, data governance, data quality, data-driven organizations, intelligent data management, Quality 4.0, quality analytics

## Abstract

Data-driven organizations increasingly depend on large, heterogeneous, and fast-moving data to sustain the quality of their products, services, and processes. The transition toward data-driven organizational quality, often discussed under the label of Quality 4.0, has so far been examined mainly through a managerial lens. As a result, the data foundations of that transition remain fragmented and under-theorized. This Conceptual Analysis reframes quality-oriented big data as a first-class data management problem and proposes a layered conceptual architecture that organizes the lifecycle of such data across five interconnected layers: multi-source acquisition, integration and fusion, data quality assurance, analytics and knowledge extraction, and data governance and ethics. The central argument is that data quality operates as a central mediating construct linking big data capability to organizational quality outcomes: analytics improves organizational quality in large part to the extent that the underlying data are accurate, complete, consistent, timely, and well governed. Drawing on the data management, data quality, and machine learning systems literatures, the article specifies the constituent elements of each layer, the dependencies among them, and the failure modes that arise when the data foundation is neglected. Five propositions and a structured research agenda position the acquisition, fusion, quality assurance, and governance of quality-oriented big data as priorities for data mining and management research. The architecture offers a data-centric vocabulary for designing and evaluating the data infrastructures on which data-driven organizational quality ultimately depends.

## 1 Introduction

When a manufacturer recalls a batch of products, a hospital audits an adverse event, or a service firm investigates a spike in complaints, the investigation is only as good as the data it can assemble. Increasingly, that data is abundant. Organizations that compete on quality now operate inside a continuous stream of it. Sensors embedded in production equipment report the state of machines and materials in near real time, execution and planning systems log every transaction and process step, inspection stations and laboratories produce structured measurement records, and customers leave dense digital traces through complaints, reviews, returns, and warranty claims (Kusiak, 2017; Tao et al., 2018; Dai et al., 2020). Taken together, these streams form a body of big data whose primary purpose is not marketing or finance but the assurance and improvement of organizational quality. In this article we call this body of data quality-oriented big data: the subset of an organization's data assets that is acquired, integrated, and analyzed in order to support decisions about the quality of products, services, and processes. Its defining feature is consequence. When it is wrong, the result is not a misdirected advertisement but a defective product shipped, a safety signal missed, or a supplier wrongly cleared.

The management literature has responded to this development with the concept of Quality 4.0, which describes the alignment of quality management practices with the technological capabilities of Industry 4.0 (Park et al., 2017; Zonnenshain and Kenett, 2020; Javaid et al., 2021; Liu et al., 2023). This stream of work has produced valuable insights into definitions and conceptual boundaries (Chiarini, 2020; Maganga and Taifa, 2023; Oliveira et al., 2025), readiness and implementation factors (Sony et al., 2020, 2021; Antony et al., 2022a), performance implications (Antony et al., 2022b; Mohsin et al., 2025), and the relationship between established excellence models and digital technologies (Carvalho et al., 2021; Fonseca et al., 2021; Asif, 2020; Dias et al., 2022). Recent systematic and bibliometric reviews confirm both the rapid growth of the field and its continuing methodological weakness: the literature remains dominated by conceptual and definitional work, with comparatively little empirical validation (Oliveira et al., 2025; Alsadi et al., 2025). What unites these contributions is a managerial vantage point: they ask how leadership, culture, skills, and strategy must change so that organizations can exploit data for quality purposes.

What this vantage point tends to take for granted is the data itself. The Quality 4.0 literature generally presumes that relevant data exist, that they can be brought together, and that they are trustworthy enough to feed analytical models. Research in data management suggests that none of these presumptions is safe. Data integration remains one of the hardest and most expensive problems in enterprise computing (Dong and Srivastava, 2015; Stonebraker and Ilyas, 2018). Machine learning pipelines in production settings fail far more often because of data problems than because of modeling problems (Sculley et al., 2015; Polyzotis et al., 2018; Breck et al., 2019). Field studies of high-stakes artificial intelligence document data cascades, in which small compromises in data work compound into serious downstream failures (Sambasivan et al., 2021). A review of big data challenges in manufacturing concludes that data issues, rather than algorithmic ones, constitute the binding constraint on quality applications (Escobar et al., 2021).

There is, in short, a gap between two literatures. The quality management literature theorizes the organizational side of data-driven quality but treats the data lifecycle as a black box. The data management literature possesses deep knowledge about acquisition, integration, quality assessment, and governance, but has rarely been asked to organize that knowledge around the specific demands of organizational quality. This article works at the junction of the two and makes three contributions. First, it defines quality-oriented big data as a distinct object of study for the data mining and management community, and specifies the profile that distinguishes it from generic enterprise analytics: consequentiality, cross-source dependence, and closed-loop operation. Second, it proposes a layered conceptual architecture that organizes the lifecycle of quality-oriented big data into five layers, multi-source acquisition, integration and fusion, data quality assurance, analytics and knowledge extraction, and data governance and ethics, and specifies for each layer its constituent elements, its dependencies, and its characteristic failure modes. Third, it advances and formalizes a central theoretical claim: that data quality is the mediating construct linking big data capability to organizational quality outcomes. This claim is expressed as five propositions, each stated so that it can in principle be falsified by firm-level or pipeline-level data, and each accompanied in the discussion by an indication of how it might be operationalized and tested.

The mediation claim is the pivot of the article and deserves a plain statement here. The prevailing intuition, encouraged by the readiness literature, is that acquiring big data capability, meaning infrastructure, tools, and skills, improves organizational quality. The claim advanced here is that a central part of this relationship runs through a specific intermediary: data quality. Capability contributes to better quality outcomes in large part because, and to the extent that, it delivers trustworthy data to the analytical layer; where the data foundation is weak, much of the capability accumulates as cost rather than converting into quality. Other mechanisms may of course operate in parallel, from improved process visibility to faster decision cycles, and the claim is not that data quality is the only path. It is that data quality is a central and often binding one, and this makes the claim a testable proposition rather than a truism: the mediation account predicts that a substantial share of the capability-to-outcome association should attenuate once data quality is introduced as an intermediate variable.

As a Conceptual Analysis, the article does not report original empirical data. Its contribution is a novel argument and model built from the published literature, in line with the purpose of this article type. The remainder proceeds as follows. Section 2 develops the background, defines the core constructs and their boundaries, positions the article against existing frameworks, and explains how the literature was selected and synthesized. Section 3 presents the architecture as a whole. Sections 4 through 8 examine each layer in turn; Section 6.1 specifies the boundary between integration and quality assurance. Section 9 develops the mediation argument, distinguishes the theoretical roles of the propositions, and discusses contributions and limitations, and Section 10 sets out the research agenda. Section 11 concludes.

## 2 Quality-oriented big data as a data management problem

### 2.1 From sampled measurement to continuous data

For most of the twentieth century, quality decisions rested on sparse, deliberately sampled measurements: control charts drawn from periodic samples, acceptance sampling of incoming lots, and audits conducted at intervals. The statistical machinery of that era was designed precisely to extract reliable conclusions from little data. The current situation inverts the problem. Instrumented processes and connected products generate observations continuously and at fine granularity, so that the scarce resource is no longer measurement but the capacity to manage and interpret measurement (Kusiak, 2017; Tao et al., 2018). The literature on manufacturing analytics documents this shift in detail, from early surveys of data mining applications in quality improvement (Köksal et al., 2011) to reviews of machine learning across production functions (Wuest et al., 2016) and of data fusion for industrial prognosis (Diez-Olivan et al., 2019).

Quality 4.0 names the organizational response to this inversion. Definitions vary, but converge on the application of Industry 4.0 technologies, including sensors, connectivity, big data, and machine learning, to the traditional aims of quality management (Javaid et al., 2021; Sader et al., 2022; Liu et al., 2023). Empirical work has mapped the motivations, barriers, and readiness factors that shape adoption (Sony et al., 2021; Antony et al., 2022a) and has begun to link adoption to organizational performance (Antony et al., 2022b). The practitioner-facing strand of this literature emphasizes that the transformation is cultural and strategic as much as technical (Park et al., 2017; Zonnenshain and Kenett, 2020).

### 2.2 Why a data management lens is necessary

A managerial account of this transformation is necessary but not sufficient, because the properties that make big data valuable are the same properties that make it difficult to manage. Volume, velocity, and variety impose engineering constraints on storage, movement, and computation (Jagadish et al., 2014; Gandomi and Haider, 2015; Sivarajah et al., 2017). Variety in particular means that quality-relevant data arrive in incompatible schemas, units, granularities, and time bases, so that the apparently simple act of asking a question across sources presupposes a substantial integration effort (Dong and Srivastava, 2015). Veracity, the dimension most directly tied to quality decisions, cannot be assumed: data quality research has shown for three decades that organizational data are routinely inaccurate, incomplete, and inconsistent, at substantial cost (Wang and Strong, 1996; Redman, 1998; Pipino et al., 2002; Batini et al., 2009; Madnick et al., 2009).

The rise of big data has renewed rather than resolved these concerns. Assessment methodologies designed for curated relational databases translate poorly to high-volume heterogeneous streams, prompting dedicated frameworks for data quality in the big data era (Cai and Zhu, 2015; Firmani et al., 2016; Taleb et al., 2021) and a growing ecosystem of measurement and monitoring tools (Cichy and Rass, 2019; Ehrlinger and Wöß, 2022). In parallel, the machine learning systems community has converged on a data-centric view of artificial intelligence, in which data collection, validation, and maintenance dominate the lifecycle cost and the failure modes of deployed models (Roh et al., 2021; Whang et al., 2023; Zha et al., 2025). These insights remain only partially integrated into the Quality 4.0 literature and have not yet been systematically organized around quality-oriented data lifecycles.

We therefore treat quality-oriented big data as a data management problem with a distinctive profile. Three features define that profile. First, consequentiality: errors propagate into physical products, service failures, and safety exposures, not merely into inaccurate reports. Second, cross-source dependence: almost every interesting quality question, from root-cause analysis to supplier evaluation, spans multiple systems and therefore depends on integration and fusion. Third, closed-loop operation: the outputs of analysis feed back into the processes that generate the data, so that the data infrastructure participates in a control loop rather than a one-way reporting pipeline. These features motivate an architectural treatment, to which we now turn.

### 2.3 Core constructs and their boundaries

Four constructs carry the argument, and the propositions depend on keeping them apart. Big data capability denotes the infrastructure, tools, and skills an organization holds for acquiring, storing, integrating, and analyzing large and heterogeneous data (Mikalef et al., 2018). It is an input and says nothing about whether the data it handles are fit for any particular use. Data quality denotes the fitness for use of the data delivered to a given analytical task, assessed along dimensions such as those in Table 1 (Wang and Strong, 1996). It is a property of data relative to a task, not of the systems that hold them, and this is what allows it to be distinguished from capability and to act as an intermediary. Data governance denotes the allocation of decision rights and accountabilities over data assets (Khatri and Brown, 2010; Abraham et al., 2019). It is a property of the organization rather than of the data, and it enters the model as a condition on how data quality is produced and maintained, not as a dimension of quality. Organizational quality outcomes denote the conformance and reliability of products, services, and processes, together with the quality decisions that shape them, such as the detection, diagnosis, and correction of non-conformities.

**Table 1:** Data quality dimensions for quality-oriented big data: definitions, characteristic failure modes, and decision-level consequences.

Dimension	Working definition	Typical failure mode in quality-oriented pipelines	Consequence for quality decisions
Accuracy	Recorded values correspond to the true state of the process or product.	Sensor drift and miscalibration accumulate unnoticed between maintenance cycles.	Control actions respond to instrument error rather than process behavior.
Completeness	All required observations and fields are present for the analytical task.	Intermittent connectivity and manual entry leave gaps concentrated in abnormal conditions.	Models underweight exactly the rare conditions that cause quality events.
Consistency	Values agree across sources, units, and system boundaries.	Parallel systems encode the same entities with conflicting codes and units.	Cross-source comparisons and supplier evaluations become non-reproducible.
Timeliness	Data are available within the latency the decision requires.	Batch transfer schedules lag the process they describe.	Real-time control degrades into after-the-fact reporting.
Validity	Values conform to defined domains, ranges, and business rules.	Schema changes upstream silently break assumptions encoded downstream.	Pipelines ingest structurally plausible but meaningless records.
Traceability	Each record carries provenance: origin, transformations, and lineage.	Fusion steps discard lineage, leaving results that cannot be audited.	Root-cause analysis stops at the dataset boundary instead of the physical cause.
Representativeness	Captured data reflect the full operating envelope of the process.	Coverage decisions favor well-instrumented lines and normal operation.	Analytics generalize confidently to conditions they have never observed.

Dimension theory follows Wang and Strong (1996), Pipino et al. (2002), and Cai and Zhu (2015).

Three boundary rules follow. Capability that does not deliver fitness for use to an analytical task does not count as data quality. Governance arrangements count only insofar as they are exercised over data assets, not merely documented. Outcomes are observed at the level of products, services, and processes, not at the level of the analytics function. Quality-oriented big data is, finally, bounded by purpose: data acquired for marketing or finance fall outside it unless they are used for quality decisions, as warranty and complaint data often are.

### 2.4 Positioning against existing frameworks

A reasonable question for any new architecture is why the existing ones do not suffice. Table 2 answers it by locating this article against the research streams and practice frameworks closest to it. Quality 4.0 research explains the organizational side of the transformation but treats the data lifecycle as a black box. Big data lifecycle and systems research specifies generic pipelines with great technical depth but without the organizational quality context that gives a quality pipeline its purpose, its genealogy problem, and its closed-loop character. Data quality frameworks define and measure fitness for use but stop short of a mechanism connecting data quality to organizational outcomes. Data governance research specifies decision rights and accountabilities but rarely binds them to a concrete analytical pipeline. The architecture proposed here is not a replacement for any of these streams; it is the connective structure that none of them supplies individually: a quality-specific data lifecycle, with an explicit outcome mechanism, under cross-cutting governance.

**Table 2:** Positioning of the proposed architecture against the closest research streams and practice frameworks.

Existing stream or framework	What it explains	What it leaves unaddressed	What this article adds
Quality 4.0 research	Leadership, culture, skills, readiness, and technology adoption for data-driven quality (Sony et al., 2021; Antony et al., 2022a).	The data lifecycle itself, which is treated as a black box.	A data-centric architecture of that lifecycle, specified layer by layer with failure modes.
Big data lifecycle and systems research	Generic acquisition, processing, and analytics pipelines and their technical challenges (Jagadish et al., 2014; Sivarajah et al., 2017).	The organizational quality context that gives the pipeline its purpose and constraints.	Quality-specific logic: product genealogy, a fusion-before-assurance precedence rule with a reconciliation loop (P2), and closed-loop feedback.
Data quality frameworks	Dimensions and assessment of fitness for use (Wang and Strong, 1996; Cai and Zhu, 2015).	A mechanism connecting data quality to organizational outcomes, and the dependence of quality on the task.	The mediation argument (P3) and task-conditional assurance (P4).
Data governance research	Decision rights and accountabilities for data assets (Khatri and Brown, 2010; Abraham et al., 2019).	Integration with a concrete quality analytics pipeline.	Governance as a cross-cutting layer that moderates the durability of the mediation path (P5).
Process models and standards	Generic stages and practices: CRISP-DM (Wirth and Hipp, 2000), TDQM (Wang, 1998), ISO/IEC 25012 (ISO/IEC, 2008), DAMA-DMBOK (DAMA International, 2017).	Which data defects matter for which quality decisions; relationships that data could contradict.	Stated relationships among layers (P1–P5), each with a role and a test (Table 3).

Table 2 also makes clear what is not new. Taken one at a time, the components of the architecture are established. Staged pipelines from data collection to modeling are codified in process models such as CRISP-DM (Wirth and Hipp, 2000) and in production machine learning platforms (Baylor et al., 2017); data quality is managed as a product lifecycle in total data quality management (Wang, 1998) and specified in standard quality models (ISO/IEC, 2008); governance and stewardship are codified in practitioner bodies of knowledge (DAMA International, 2017); and feedback from analysis to data collection is familiar from all of them. Read critically, these frameworks share three limitations for the present purpose. They are domain-neutral, so they do not say which data defects matter for which quality decision. They treat data quality as a stage or a list of generic dimensions, rather than as a variable whose level depends on upstream integration and whose effect depends on the analytical task. And they prescribe what should be done without stating relationships that data could contradict.

The contribution of this article therefore lies less in its components than in four relationships among them. The first is a precedence rule between fusion and assurance: the semantic and temporal soundness of integration is a precondition that assurance cannot establish on its own, which creates a lineage obligation from Layer 2 to Layer 3 and a reconciliation loop between them (Section 6.1; P2). The second is a task-conditional mapping in which specific quality dimensions bind specific quality decisions, instead of a uniform quality threshold (Table 1; P4). The third is a mediation mechanism in which data quality carries a substantial part of the effect of capability on organizational quality outcomes (P3). The fourth is a moderation claim in which governance determines the durability of that mechanism rather than its existence (P5). The layering is a means of stating these relationships precisely; it is the relationships, not the layers, that are offered as new and that the propositions put to test.

### 2.5 Approach and literature selection

The article follows the model-building design for conceptual papers, which develops a framework that predicts relationships between constructs (Jaakkola, 2020), and builds that framework by identifying, integrating, and synthesizing concepts from several disciplinary literatures (Jabareen, 2009). The literature was selected purposively rather than through a systematic search. For each layer and each construct, we drew on foundational works that established the concept, recent reviews and surveys that summarize its current state, and empirical studies that bear directly on the relationships proposed. Sources came from five streams: Quality 4.0 and quality management; big data and data integration; data quality; machine learning systems; and data governance and ethics. Works were included when they addressed data-related conditions of analytical or quality outcomes, and excluded when they addressed quality management without reference to data, or big data without reference to its organizational use. This design suits theory building but does not guarantee complete coverage, a limitation discussed in Section 9.4.

## 3 A layered conceptual architecture: overview

Figure 1 presents the proposed architecture. It organizes the lifecycle of quality-oriented big data into five layers. Four of them form a vertical value chain: multi-source acquisition (Layer 1), integration and data fusion (Layer 2), data quality assurance (Layer 3), and analytics and knowledge extraction (Layer 4). The fifth layer, data governance and ethics, is deliberately drawn as a cross-cutting spine rather than a stage: governance decisions constrain and enable every other layer, from what may be collected to how model outputs may be used.

**Figure 1:**
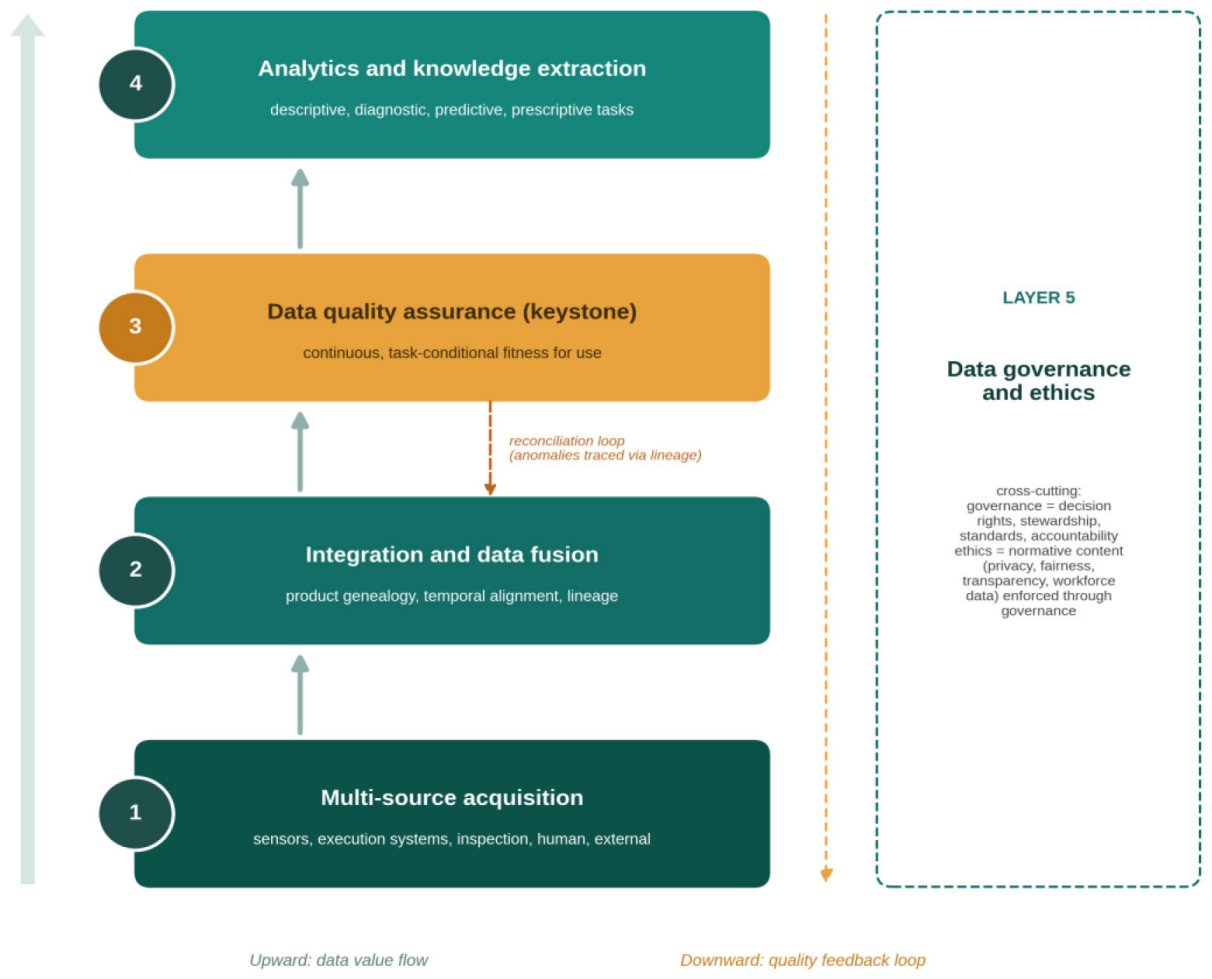
The layered conceptual architecture for managing quality-oriented big data. Four layers form an upward value flow from acquisition to analytics; data governance and ethics operate as a cross-cutting spine; analytical insight returns downward as a quality feedback loop. Data quality assurance (Layer 3) is highlighted as the keystone layer, and the dashed arrow between Layers 3 and 2 marks the reconciliation loop through which anomalies detected in assurance are traced back to fusion (Section 6.1). Within Layer 5, ethics supplies the normative content and governance the means of enforcing it (Section 8).

The architecture should be read through two flows. The upward flow is the value flow: raw signals acquired at Layer 1 gain meaning through fusion at Layer 2, gain trustworthiness through assurance at Layer 3, and gain decision relevance through analytics at Layer 4. The downward flow is the feedback flow: analytical findings redirect acquisition (new sensors, new sampling priorities), refine fusion (new linkage keys, corrected alignments), and recalibrate assurance (new validation rules learned from failures). The two flows together make the architecture a loop, consistent with the closed-loop character of quality work.

Two structural claims follow from the layering. The first is a dependency claim: each layer consumes guarantees produced by the layer below it and produces guarantees for the layer above it. Analytics presupposes assured data; assurance presupposes integrated data; integration presupposes acquired data. Defects therefore propagate upward, and a weakness in a lower layer silently caps the value of every investment above it, a pattern documented empirically as data cascades (Sambasivan et al., 2021) and as hidden technical debt (Sculley et al., 2015). The second is a keystone claim: Layer 3, data quality assurance, occupies the structurally decisive position, because it is the point at which the pipeline either does or does not convert integrated data into trustworthy data. Section 9 develops this claim into the article's central proposition.

### 3.1 A worked illustration: tracing a field failure

To make the architecture concrete, consider a stylized but representative task: an organization observes an unexpected rise in field failures of a component and wants to know why. The question traverses the architecture from top to bottom and back. At the analytics layer, a diagnostic model looks for process conditions associated with failed units. That model can only ask its question of data that the fusion layer has linked, connecting each failed unit to the machine, batch of raw material, process parameters, operator, and ambient conditions under which it was made, an entity-resolution task across systems that were never designed to share keys. The linkage in turn is only as trustworthy as the assurance layer allows: if timestamps from two source systems are misaligned, the model may associate a failure with the wrong process window and confidently indict an innocent parameter. The assurance layer can only check data that the acquisition layer captured: if the suspect condition occurs on an uninstrumented line, or was filtered out as an outlier at capture, no downstream analysis can recover it. And every one of these steps is bounded by governance: whether the linkage keys exist, whether the validation rules are maintained, and whether operator-level data may be used at all. The same trace runs in reverse as a feedback loop: the finding tells the organization which line to instrument next, which linkage to formalize, and which validation rule to add. This single example exercises all five layers, both flows, and the dependency and keystone claims, and it is the pattern the rest of the article generalizes.

## 4 Layer 1: multi-source acquisition

The base layer concerns what enters the pipeline at all. Quality-relevant data originate in at least five families of sources: machine and sensor data from instrumented equipment and connected products; process execution data from manufacturing execution, enterprise resource planning, and workflow systems; inspection and laboratory data from measurement stations; human-generated records such as operator logs, non-conformity reports, and audit findings; and external data from suppliers, logistics partners, and customers (Wuest et al., 2016; Tao et al., 2018; Dai et al., 2020). The families differ sharply in volume, structure, latency, and reliability, and a central task of this layer is to make those differences explicit rather than to flatten them.

Acquisition is a design problem, not a passive recording problem. Three design decisions carry particular weight for downstream quality analytics. The first is coverage: which processes, machines, and product families are instrumented, and which remain dark. Coverage decisions create the blind spots within which quality problems can develop unobserved, and they are rarely revisited once made. The second is representativeness: whether the captured data reflect the full operating envelope of the process, including rare but consequential conditions. The machine learning literature is unambiguous that collection-stage bias is among the most damaging and least visible defects a dataset can carry, because no downstream procedure can recover information that was never captured (Roh et al., 2021; Whang et al., 2023). The emerging literature on data readiness for artificial intelligence formalizes exactly this concern, proposing systematic assessments of whether captured data are fit to support downstream models before those models are built (Hiniduma et al., 2025). The third is metadata at capture: recording, at acquisition time, the provenance, calibration state, and context of each stream. The practice of documenting datasets systematically, argued for machine learning corpora under the name of datasheets, applies with equal force to industrial data streams whose measurement context otherwise evaporates (Gebru et al., 2021).

The characteristic failure modes of this layer follow directly: blind spots from narrow coverage, silent bias from unrepresentative capture, and orphaned data whose meaning is unrecoverable because context was not recorded. Each failure is invisible at the point where it is committed and expensive at the point where it is discovered, which is typically in an analytical result that cannot be trusted or explained. This asymmetry between the cost of prevention and the cost of discovery is a recurring pattern across the architecture, first documented at scale in production machine learning systems (Sculley et al., 2015; Polyzotis et al., 2018).

## 5 Layer 2: integration and data fusion

The second layer converts many streams into one coherent body of evidence. Its scientific foundations lie in two adjacent traditions. Data integration research addresses schema heterogeneity, entity resolution, and record linkage across enterprise systems, and its central lesson is sobering: integration at scale remains labor-intensive, error-prone, and among the dominant cost items of any serious analytics program (Dong and Srivastava, 2015; Stonebraker and Ilyas, 2018). Data fusion research, with roots in multi-sensor systems, addresses the combination of overlapping and partially conflicting signals into estimates that are more accurate than any single source, across signal, feature, and decision levels (Khaleghi et al., 2013). Machine learning has broadened fusion methodology considerably in recent years (Meng et al., 2020), and industrial applications increasingly rely on fused sensor and process data for prognosis and condition assessment (Diez-Olivan et al., 2019).

For quality-oriented big data, two integration tasks stand out. The first is product genealogy: linking every unit of output to the materials, machines, parameters, operators, and environmental conditions that produced it, across systems that were never designed to share keys. Genealogy is the backbone of root-cause analysis, recall management, and supplier quality, and it is an entity-resolution problem of considerable difficulty. The second is temporal alignment: placing measurements from sources with different clocks, sampling rates, and latencies onto a common time base. Quality analytics is intensely temporal, because cause precedes effect along a process flow, and misaligned time bases manufacture spurious lead-lag relationships.

The failure modes of this layer are correspondingly analytical rather than merely operational. Incorrect entity resolution attributes outcomes to the wrong units, machines, or suppliers. Temporal misalignment produces pseudo-signals: correlations that exist in the integrated dataset but not in the physical process. Both defects pass silently through statistical checks, because the integrated data are internally well formed; they are simply wrong about the world. This is why the architecture treats fusion quality as a precondition for, rather than a component of, data quality assurance: Layer 3 can verify that data are consistent and complete, but it cannot detect that a join was semantically wrong unless lineage from Layer 2 is preserved and inspectable. Section 6.1 states the rule that separates the two layers and the feedback expected between them.

## 6 Layer 3: data quality assurance

The third layer is the keystone of the architecture. Data quality research defines quality as fitness for use and decomposes it into dimensions such as accuracy, completeness, consistency, and timeliness, established through foundational empirical work with data consumers (Wang and Strong, 1996) and operationalized through measurement methodologies (Pipino et al., 2002; Batini et al., 2009; Madnick et al., 2009), with dimension theory still being actively refined (Haug, 2025). The costs of neglect are well documented at the enterprise level (Redman, 1998) and in analytics-intensive settings specifically (Hazen et al., 2014). The big data era has forced a rethinking of both dimensions and methods: assessment must now operate on heterogeneous, high-velocity, weakly structured data, often without a single authoritative reference copy (Cai and Zhu, 2015; Firmani et al., 2016), which has produced holistic frameworks for continuous quality management of big data (Taleb et al., 2021), comparative overviews of assessment frameworks (Cichy and Rass, 2019), and a substantial tool ecosystem for measurement and monitoring (Ehrlinger and Wöß, 2022).

Two shifts distinguish assurance in this architecture from traditional data cleansing. The first is from episodic to continuous. In a closed-loop setting fed by streams, quality assurance cannot be a project conducted before analysis; it must be a monitoring function that runs alongside the pipeline, validating new data against expectations and raising alarms on drift, in the manner of data validation systems developed for production machine learning (Breck et al., 2019; Polyzotis et al., 2018). The second is from generic to task-conditional. Fitness for use is relative to the use: a real-time process control application is exquisitely sensitive to timeliness and far more tolerant of isolated gaps, while a model trained to predict field failures is sensitive to completeness and representativeness across months of history and indifferent to millisecond latency. Assurance therefore requires an explicit mapping from analytical tasks to the quality dimensions that bind them.

Table 1 provides that mapping in condensed form. For each of seven dimensions relevant to quality-oriented big data, it states a working definition, the typical mechanism by which the dimension degrades in multi-source quality pipelines, and the decision-level consequence. The table makes visible the argument that runs through this article: every row terminates in a quality decision that is degraded, delayed, or wrong. Data quality is not an information technology housekeeping concern; it is the transmission mechanism through which data infrastructure does or does not reach organizational outcomes. Empirical support for this transmission view exists: firm-level evidence indicates that the contribution of big data to decision quality is conditional on data quality and the diagnosticity of the data for the decision at hand (Ghasemaghaei and Calic, 2019), and case evidence on big data decision processes identifies data quality among the decisive process factors (Janssen et al., 2017).

### 6.1 The boundary between integration and quality assurance

Because fusion and assurance both concern whether data can be trusted, their boundary needs an explicit rule. The rule adopted here rests on where a defect originates and what is needed to detect it. A problem belongs to Layer 2 when it arises from how records from different sources are related, through schema mapping, entity resolution, or temporal alignment, and can be detected only with knowledge of the correspondence between sources. A problem belongs to Layer 3 when it concerns the fitness of values or records for a given use and can be detected against domain rules, reference values, or expected distributions, regardless of how the records were linked. A simple test follows: if the defect would still exist were each source used on its own, it belongs to Layer 3, or to Layer 1 if it was introduced at capture; if it exists only because sources were combined, it belongs to Layer 2. A wrongly matched batch identifier is therefore a Layer 2 defect, whereas a drifted sensor reading is a Layer 3 defect even when it appears in fused data.

The two layers are nevertheless not a one-way sequence. Assurance often detects symptoms whose cause lies in fusion, such as implausible lead-lag patterns or units that appear to have been produced on two lines at once. The architecture therefore expects an iterative reconciliation loop: Layer 3 flags the anomaly, lineage preserved by Layer 2 allows it to be traced to a specific linkage or alignment step, and the corrected output is reassessed. Fusion in turn depends on assurance for its inputs, since entity resolution on inaccurate keys fails. The ordering in Figure 1 describes the dominant direction of dependence, not the absence of feedback, and the figure marks the reconciliation loop explicitly.

## 7 Layer 4: analytics and knowledge extraction

The fourth layer converts assured data into decisions and knowledge. Its content is best organized by task rather than by algorithm, in four families of ascending ambition. Descriptive tasks make the current state of quality visible through monitoring and reporting across integrated sources. Diagnostic tasks explain deviations, extending classical root-cause analysis with data mining over genealogy and process data; this family has the longest research pedigree in quality applications (Köksal et al., 2011). Predictive tasks anticipate quality events before they occur, including defect prediction from process signatures and failure prognosis from fused condition data (Wuest et al., 2016; Diez-Olivan et al., 2019; Escobar et al., 2021). Prescriptive tasks close the loop by recommending or executing interventions, from parameter adjustment to the prioritization of non-conformities; work on risk-based prioritization of non-conformity handling illustrates how analytical structure can be brought to a traditionally judgment-driven quality process (Ravoy and Parmet, 2021).

The systems perspective matters as much as the task taxonomy. Production experience with machine learning demonstrates that models are a small fraction of a deployed analytical system, surrounded by data collection, verification, feature management, and monitoring infrastructure in which most failures and most maintenance costs reside (Sculley et al., 2015; Polyzotis et al., 2018). For quality applications, three systems requirements deserve emphasis. Interpretability: quality engineers must act on model outputs in the physical world, and an unexplainable alarm competes poorly with an explainable one. Drift management: processes, materials, and products change, so models degrade by default and require monitored retraining. Feedback capture: every intervention triggered by the analytics layer generates new data about its own effectiveness, and a pipeline that fails to capture this closes the loop on paper but not in fact.

The failure modes of this layer are subtle because they masquerade as success. A model can achieve excellent accuracy on data that Layer 1 captured unrepresentatively, reproduce a pseudo-signal that Layer 2 manufactured, or silently decay as reality drifts away from its training distribution. In each case the defect originates below and merely surfaces here, which is the practical content of the dependency claim of Section 3: analytical sophistication cannot compensate for foundational weakness, a conclusion that the data-centric turn in machine learning has reached independently (Roh et al., 2021; Whang et al., 2023).

## 8 Layer 5: data governance and ethics

The fifth layer answers a different kind of question: not what can be done with quality-oriented big data, but what may and should be done, by whom, and under whose accountability. Data governance research frames this as the specification of decision rights and accountabilities for data assets, covering decision domains such as quality standards, access, lifecycle, and metadata (Khatri and Brown, 2010), and has matured into structured frameworks and research agendas at the organizational level (Abraham et al., 2019). Recent work ties governance directly to the trustworthiness of artificial intelligence, arguing that trustworthy analytical systems presuppose governed data (Janssen et al., 2020).

Governance earns its cross-cutting position in the architecture for a structural reason: it is the only layer whose outputs are constraints and mandates for all the others. Stewardship assignments determine who is accountable for the coverage decisions of Layer 1. Standards for identifiers and metadata determine whether the genealogy problem of Layer 2 is tractable. Ownership of validation rules determines whether the monitoring of Layer 3 is maintained after its champions move on. Approval and audit requirements determine which models of Layer 4 may act on the process, and under what human oversight. Where governance is weak, each layer optimizes locally and the architecture degrades into disconnected projects; the durability of the whole pipeline, rather than its initial construction, is what governance protects.

Ethics enters this layer through the specific character of quality-oriented data. Process data are also people data: operator identifiers, shift patterns, and performance traces are embedded in the same streams that describe machines and materials, so quality analytics borders on workforce surveillance and must be designed deliberately to respect that border (Floridi and Taddeo, 2016). The global convergence of artificial intelligence ethics guidance on principles of transparency, justice, responsibility, and privacy provides a usable scaffold (Jobin et al., 2019), and gives quality organizations a concrete design obligation: analytical uses of person-linked process data require explicit purpose limits, aggregation defaults, and worker visibility into what is collected and why. In this architecture, ethical review is not an external checkpoint but a governance function exercised over acquisition scopes, fusion linkages, and analytical uses alike.

Placing ethics within the governance layer, rather than drawing it as a separate dimension, requires justification, because the two are conceptually distinct. Governance concerns who decides and who is accountable: decision rights, stewardship, standards, and accountability. Ethics concerns what ought to be decided: privacy, fairness, transparency, and the limits of workforce monitoring. The architecture keeps this distinction and nests ethics within Layer 5 for a functional reason. Ethical principles on their own do not change what a pipeline does; they take effect only when translated into decision rights, review procedures, and accountable roles (Mittelstadt, 2019). Governance is the mechanism through which such principles reach each layer: purpose limits constrain acquisition, linkage approvals constrain fusion, and use approvals constrain analytics. Ethics thus supplies the normative content of Layer 5, and governance the means of enforcing it.

The nesting is not a claim that ethics reduces to governance or to compliance. Ethical concerns can arise where no governance rule applies, and a governance regime can operate well and still be ethically deficient, for example when it formally authorizes intrusive monitoring. An alternative design would treat ethics as a second cross-cutting dimension, evaluated independently of governance. We retain the nested form because it ties each ethical obligation to an accountable decision, and we note the alternative in Section 9.4 as a direction for comparative work.

## 9 Discussion

### 9.1 Data quality as the mediating construct

The layered analysis converges on one argument, depicted in Figure 2. Investments in big data capability, understood as the combination of infrastructure, tools, and skills that the analytics capability literature has studied extensively (Mikalef et al., 2018), reach organizational quality outcomes in large part through an intermediary rather than wholly through a direct effect. A central part of their effect travels through the quality of the data that the pipeline delivers to its analytical layer. When acquisition is representative, fusion is sound, and assurance is continuous, capability is converted into trustworthy analytical input and thence into better quality decisions; when they are not, much of it accumulates as cost. Other channels, such as improved process visibility, faster decision cycles, or organizational learning, may contribute in parallel, and the argument here is not that they are absent but that the data quality channel is central and frequently binding. This mediation logic is consistent with firm-level evidence that data quality and data diagnosticity condition the decision benefits of big data (Ghasemaghaei and Calic, 2019), with process evidence on big data decision-making quality (Janssen et al., 2017), and with the broader finding that the productivity effects of data-driven decision making are contingent on complementary practices rather than automatic (Brynjolfsson and McElheran, 2016).

**Figure 2:**
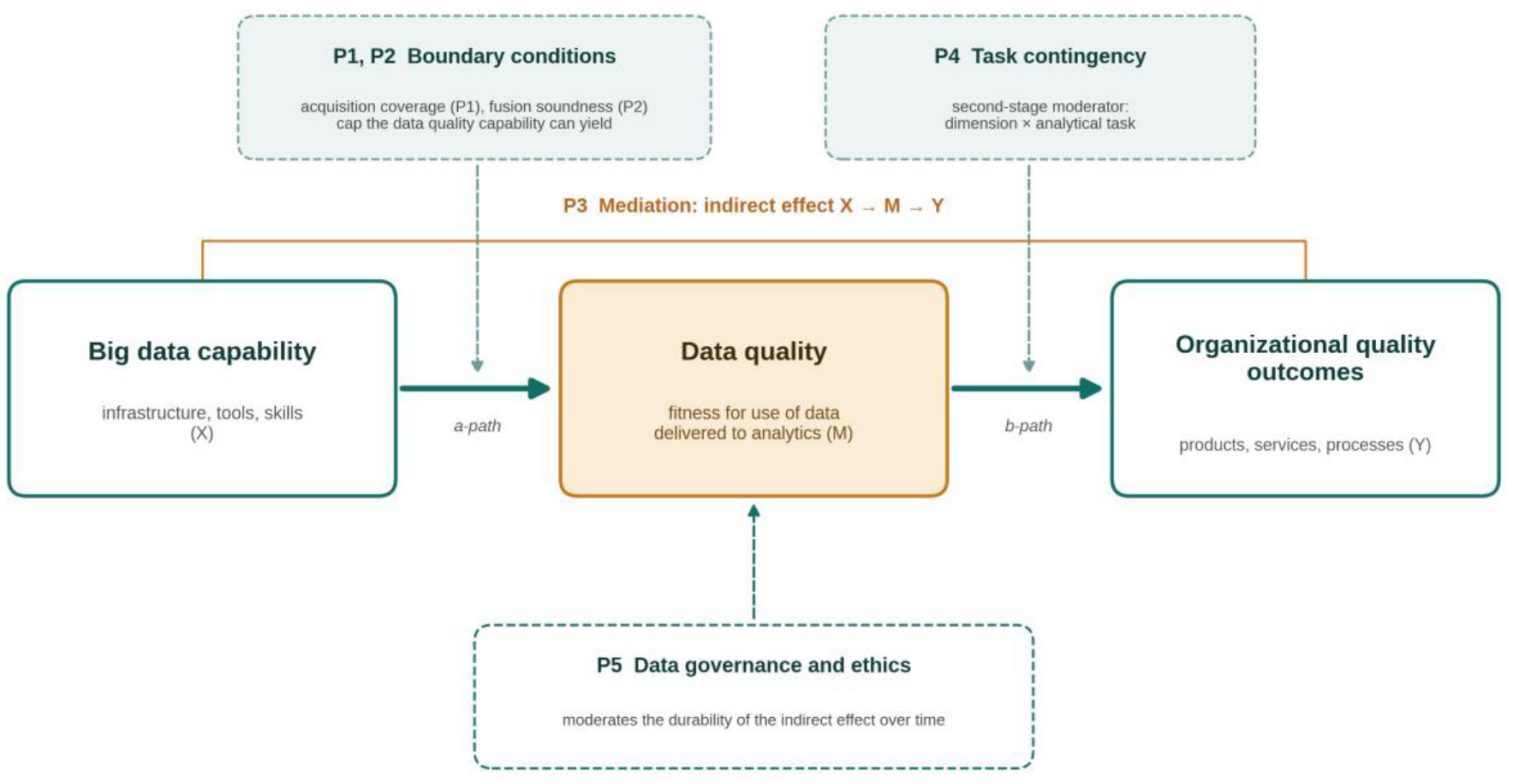
The central theoretical claim and the roles of the five propositions. Big data capability **(X)** reaches organizational quality outcomes **(Y)** through data quality **(M)**; the indirect effect is the mediation hypothesis (P3). Acquisition coverage and fusion soundness act as boundary conditions on the first stage (P1, P2), analytical task moderates the second stage (P4), and data governance and ethics moderate the durability of the indirect effect over time (P5).

Five propositions formalize the argument at the level of the architecture, and Table 3 states them compactly with their theoretical role and architectural locus. They play different theoretical roles, and Figure 2 and Table 3 distinguish these roles explicitly because each implies a different statistical test (Baron and Kenny, 1986; Hayes, 2018). P1 and P2 are boundary conditions on the first stage of the model, the path from capability to data quality: acquisition coverage and representativeness (P1) and the semantic and temporal soundness of fusion (P2) set an upper limit on the data quality that capability can yield, and can be modeled as first-stage moderators or as ceiling constraints. P3 is the central mediation hypothesis: capability affects organizational quality outcomes in substantial part through data quality. P4 is a moderation hypothesis on the second stage, the path from data quality to outcomes: the effect of a given quality dimension depends on the analytical task, which is an interaction between dimension and task, so assurance must be conditioned on the task portfolio rather than applied uniformly. P5 is a moderation hypothesis over time: governance does not create the indirect path but determines whether it persists as systems, staff, and products change. Taken together, the propositions describe a mediation model that is bounded at its first stage, moderated at its second, and sustained by governance, a structure that conditional process analysis can estimate (Hayes, 2018). Together the propositions convert the architecture from a descriptive map into a testable theory.

**Table 3:** Propositions derived from the architecture, with their theoretical roles.

**No**.	**Proposition**	Theoretical role	Architectural locus
P1	The quality-analytical value obtainable at Layer 4 is bounded above by the coverage and representativeness of acquisition at Layer 1; no downstream method recovers information that was never captured.	Boundary condition on the first stage (capability → data quality)	Layer 1
P2	The validity of cross-source quality inferences is conditional on the semantic and temporal soundness of integration and fusion; entity misresolution and time-base misalignment propagate as pseudo-signals indistinguishable from findings.	Boundary condition on the first stage (capability → data quality)	Layer 2
P3	Data quality is a central mediating mechanism through which big data capability contributes to organizational quality outcomes; a substantial part of the effect of capability investments on outcomes operates through the trustworthiness of the data delivered to analytics.	Mediation (indirect effect X → M → Y)	Layer 3 (keystone)
P4	Data quality dimensions bind analytical tasks differentially: timeliness binds real-time control, completeness and representativeness bind prediction, and traceability binds diagnosis; assurance must therefore be task-conditional.	Moderation of the second stage (dimension × task)	Layers 3–4
P5	Data governance moderates the durability of the capability-to-outcome path: under weak governance the mediation established by P3 decays as systems, staff, and products change.	Moderation of the indirect effect over time	Layer 5

The shaded row marks the central mediation claim (P3).

Because the propositions are meant as a testable theory rather than a metaphor, it is worth indicating how each could be confronted with evidence. P3, the mediation claim, is directly amenable to the mediation and moderated-mediation designs already used in the analytics-capability literature (Mikalef et al., 2018; Ghasemaghaei and Calic, 2019): measure big data capability, a data quality index, and organizational quality outcomes across firms, and test whether the capability-to-outcome association attenuates when data quality is included as an intermediate variable. P1 and P2, as boundary conditions, are testable within firms by relating acquisition coverage and fusion soundness, both measurable at the pipeline level, to the realized accuracy of downstream quality models, holding the model constant. P4, as a second-stage moderator, predicts an interaction: the marginal effect of improving a given quality dimension on decision quality should depend on the analytical task, which can be examined experimentally by degrading specific dimensions and observing task-specific performance loss. P5, as a moderator over time, predicts that the mediation of P3 weakens over time or under organizational change where governance is weak, which longitudinal or multi-case designs can probe. Stating the predictions in this form is what distinguishes the architecture from a descriptive taxonomy: each layer yields a claim that data could contradict. Any test of P3 must also rule out two rival explanations: firms with better quality outcomes may invest more in data work, reversing the direction of effect, and a common cause such as quality culture may drive both data quality and outcomes. Longitudinal designs, instrumental approaches, and comparisons of lines within the same firm can address these threats (Antonakis et al., 2010).

### 9.2 Contributions to data mining and management research

For the data mining and management community, the article makes three offers. First, it delineates a coherent application domain, quality-oriented big data, whose profile of consequentiality, cross-source dependence, and closed-loop operation differentiates it from generic enterprise analytics and gives familiar problems a distinctive form: entity resolution becomes product genealogy, stream validation becomes process-aware monitoring, and fairness questions acquire a workforce dimension. Second, the layered architecture supplies a shared vocabulary in which contributions from separate subfields, integration, fusion, quality assessment, machine learning systems, and governance, can be located relative to one another and to the decisions they ultimately serve. Third, the propositions identify where empirical work is most needed, in particular measurement of the mediation path (P3) and of the differential criticality of quality dimensions across analytical tasks (P4), questions on which the data management field holds the methodological keys.

### 9.3 Implications for practice

For organizations, the architecture suggests a different investment logic. Because the architecture has not been empirically validated, this logic should be read as a recommendation derived from the framework, not as an established finding. The prevailing pattern, visible in the readiness literature, is to begin at Layer 4 by procuring analytics and then to discover Layers 1 through 3 as obstacles (Sony et al., 2021; Antony et al., 2022a). If the propositions hold, organizations would be better served by the reverse sequence: auditing acquisition coverage and metadata practice first; making genealogy and temporal alignment explicit engineering objects second; instituting continuous, task-conditional quality monitoring third; and letting the analytical portfolio grow at the pace the assured foundation supports. Whether this sequence produces better outcomes than the alternatives is itself an empirical question, and organizations with mature analytics may reasonably follow a different path. The keystone position of Layer 3 also suggests a concrete management artifact: a register linking each analytical use case to the quality dimensions that bind it, with monitored thresholds, which would operationalize fitness for use at the level of the portfolio.

### 9.4 Limitations

The argument has six limitations, which also define the conditions under which it should be read. First, the contribution is conceptual and awaits empirical test. The propositions are stated so as to be falsifiable, but none is validated here, and the architecture has not been evaluated with practitioners or applied in an organization. Statements about preferable investment sequences are therefore propositions, not findings.

Second, the literature was selected purposively (Section 2.5). This design suits theory building, but it may favor widely cited and English-language work and may miss relevant studies from practitioner and regional literatures.

Third, most examples and much of the supporting evidence come from manufacturing. The architecture is intended to apply to services and healthcare as well, but data sources, the meaning of genealogy, and the relevant outcomes differ in those settings, so transfer should be examined rather than assumed.

Fourth, the architecture assumes a degree of organizational integration that many firms, particularly smaller ones, do not have. The layer logic still applies, but the appropriate scale, tooling, and governance differ, and these boundary conditions deserve study.

Fifth, the layered ordering is one defensible design among several. Governance could be placed as the base layer, fusion and assurance could be merged, and ethics could be treated as a cross-cutting dimension independent of governance (Section 8). The layers also interact iteratively (Section 6.1), so the linear depiction simplifies practice. Comparative conceptual and empirical work would show which design best predicts outcomes.

Sixth, testing the mediation claim raises problems of measurement and identification. Data quality is hard to measure consistently across firms, capability and data quality are often reported by the same respondents, and reverse causality or common causes may produce the predicted associations without the proposed mechanism (Antonakis et al., 2010). Pipeline-level measures and longitudinal designs can reduce these threats but not remove them.

These limitations mark the edge of what a Conceptual Analysis can claim, and the beginning of the agenda that follows.

## 10 A research agenda for data mining and management

Table 4 organizes the agenda by layer. The unifying thread is that each layer poses problems that are simultaneously scientifically substantive for data mining and management and decision-critical for organizational quality. Three lines deserve emphasis. The first is quality-aware acquisition: methods that decide, adaptively and under budget constraints, what to sense and record next in order to maximize downstream quality-analytical value, extending active data collection ideas (Roh et al., 2021) into industrial settings. The second is verifiable fusion: genealogy and alignment techniques that carry proofs or diagnostics of their own semantic soundness, so that Layer 3 can audit Layer 2 rather than trust it. The third is task-conditional quality assurance at stream scale: monitoring frameworks that operationalize fitness for use per analytical task, integrating validation-system engineering (Breck et al., 2019) with quality-dimension theory (Wang and Strong, 1996; Cai and Zhu, 2015) and the emerging tool ecosystem (Ehrlinger and Wöß, 2022).

**Table 4:** A layer-by-layer research agenda for data mining and management on quality-oriented big data.

Layer	Open problems for data mining and management	Illustrative research questions
1. Acquisition	Quality-aware, budget-constrained adaptive sensing; metadata capture standards for industrial streams.	How should an organization decide, adaptively, what to sense next to maximize downstream quality-analytical value?
2. Fusion	Verifiable product genealogy; temporal alignment with soundness diagnostics; lineage-preserving fusion at stream scale.	Can fusion procedures carry machine-checkable evidence of their own semantic correctness?
3. Assurance	Task-conditional fitness-for-use monitoring; drift detection tuned to process semantics; quality-dimension metrics for weakly structured data.	Which quality dimensions bind which analytical tasks, and with what measurable thresholds?
4. Analytics	Interpretable prediction for physical intervention; feedback-capturing closed-loop learning; maintenance-cost-aware model portfolios.	How should closed-loop quality analytics capture the effects of its own interventions as training signal?
5. Governance	Stewardship models for cross-cutting data assets; auditability requirements for autonomous quality actions; workforce-data ethics in process analytics.	What governance arrangements keep the capability-to-outcome path stable through organizational change?

## 11 Conclusion

The movement toward data-driven organizational quality has been narrated, so far, largely as a management story. This article has argued that it is equally, and foundationally, a data management story. It has defined quality-oriented big data as a distinct object of study: the body of heterogeneous, multi-source, continuously generated data that organizations acquire, integrate, and analyze in order to decide about the quality of their products, services, and processes. It has organized the lifecycle of that data into a five-layer conceptual architecture, in which multi-source acquisition, integration and fusion, data quality assurance, and analytics form an upward value chain, governance and ethics operate as a cross-cutting spine, and analytical insight returns downward as a feedback loop. Read this way, the key questions about data-driven quality change character: not whether organizations are culturally ready for the transformation, but whether their acquisition is representative, their fusion semantically sound, their data quality continuously and task-conditionally assured, their analytics engineered as systems rather than as models, and their governance durable enough to hold the whole together.

The theoretical core of the argument is the mediation claim. Big data capability reaches organizational quality outcomes in large part through the quality of the data delivered to the analytical layer, a path that is bounded upstream by acquisition and fusion, differs by analytical task, and persists only where governance sustains it. Data quality is not proposed as the sole mechanism, but as a central and frequently binding one. The five propositions state this claim in falsifiable form, and the layered agenda of Section 10 indicates where the data mining and management community is best placed to test and extend it: quality-aware acquisition, verifiable fusion, task-conditional assurance at stream scale, closed-loop analytics, and governance arrangements for cross-cutting data assets. For practitioners, the same logic suggests, pending empirical validation, building upward from the data foundation rather than downward from purchased analytics, and it points to a concrete management artifact in the form of a register that links each analytical use case to the quality dimensions and monitored thresholds that bind it.

Two developments make this reframing timely rather than optional. Within machine learning, the data-centric turn has established that the returns to further modeling sophistication are increasingly limited by the state of data work (Sambasivan et al., 2021; Whang et al., 2023). Within quality management, the maturing Quality 4.0 literature is moving from conceptualization toward implementation and performance evidence (Antony et al., 2022b; Liu et al., 2023), which is precisely the stage at which data foundations begin to bind. As quality decisions become more automated and more consequential, the trustworthiness of the data beneath them stops being a technical detail and becomes part of the substance of quality itself. If the central claim of this article survives empirical scrutiny, it would imply that better managed data is among the most direct paths to better organizational quality.
